# Genomics-Driven Discovery of Plantariitin A, a New Lipopeptide in *Burkholderia plantarii* DSM9509

**DOI:** 10.3390/molecules30040868

**Published:** 2025-02-14

**Authors:** Xiuling Wang, Zhuo Zhang, Jun Fu, Ruijuan Li

**Affiliations:** State Key Laboratory of Microbial Technology, Shandong University, Qingdao 266237, China; 18706380569@163.com (X.W.); sduzhangzhuo@163.com (Z.Z.)

**Keywords:** *Burkholderia plantarii*, lipopeptides, recombineering system, in situ activation, unusual amino acid

## Abstract

A significant number of silent biosynthetic gene clusters (BGCs) within the *Burkholderia* genome remain uncharacterized, representing a valuable opportunity for the discovery of new natural products. In this research, the recombineering system ETh1h2e_yi23, which facilitates recombination in *Burkholderia* and was developed in our previous study, was used for mining the BGCs of *B. plantarii* DSM9509. By using this recombineering system, the constitutive promoter was precisely inserted into the genome, resulting in the activation of the silent *pla* BGC, which led to the production of a new lipopeptide named plantariitin A. A distinctive characteristic of this lipopeptide is the incorporation of a non-proteinogenic amino acid residue, i.e., amino-1,2,3,6-tetrahydro-2,6-dioxo-4-pyrimidinepropanoic acid (ATDPP), which has not been identified in other natural products. A biological activity assay demonstrated that plantariitin A exhibits anti-inflammatory activity. This study further substantiates the notion that the in situ activation of silent BGCs is a crucial strategy for the discovery of new natural products within the genus *Burkholderia*. With the increasing availability of genomic data and the development of bioinformatics tools, *Burkholderia* is poised to emerge as a prominent source for the development of new lipopeptides.

## 1. Introduction

Natural products and their derivatives from microorganisms, such as antitumor spliceostatins [1], antibacterial compound thailandamides, antifungal aminopyrrolnitrin [2], and the anticancer drug romidepsin [3], approved by the Food and Drug Administration (FDA), play significant roles in various aspects of human health and agriculture [4]. Lipopeptides (LP_S_), as a prominent class of natural products, exhibit a wide range of biological activities due to their structural diversity [5,6]. Structurally, LPs are characterized by a peptide composed of amino acids that are covalently linked to a fatty acid chain. The variation in the type and quantity of amino acids within the peptide chain, along with alterations in the length and degrees of unsaturation of the fatty acid chain, result in the emergence of various functional LPs.

Studies have shown that amino acid residues, particularly nonproteinogenic amino acids, significantly influence the bioactivity of LPs as fundamental components of peptides. For instance, daptomycin, an acidic LP comprising 13 amino acid residues including L-ornithine (Orn), L-kynurenine (Kyn), and L-3-methylglutamate, demonstrated a marked reduction in bactericidal activity against *Bacillus subtilis* PY79 when Kyn was replaced with tryptophan (Trp) [7]. In addition, nonproteinogenic amino acids represent a vital source of drugs. L-4-chlorokynurenine (L-4-Cl-Kyn) is a neuropharmaceutical drug candidate, demonstrating efficacy in animal models for the treatment of neuropathic pain, epilepsy, and Huntington’s disease, and is currently under development for addressing major depression [8]. In nature, L-4-Cl-Kyn is found as a nonproteinogenic amino acid residue in the LP antibiotic taromycin, and its biosynthesis, which involves a three-step enzyme pathway from L-Trp to L-4-Cl-Kyn, has been elucidated through the investigation of the taromycin BGC [8]. The incorporation of nonproteinogenic amino acids, synthesized via various enzymes such as hydroxylases, dehydrogenases, aminotransferases, and P450 monooxygenases, enhances the diversity of LPs [9].

In general, non-ribosomal peptide synthetases (NRPSs) are involved in the biosynthesis of peptide chains on LPs [10,11]. These enzymes are characterized as multimodular, with each module responsible for the incorporation of a single amino acid residue into the peptide backbone. Each module comprises a series of conserved catalytic domains including an adenylation (A) domain [12], a thiosylated (T) domain or a peptidyl carrier protein (PCP), a condensation (C) domain, and a thioesterase (TE) domain [13,14]. Fatty acid chains are produced by fatty acid synthases or polyketide synthases (PKSs) [15], PKS is also a modular enzyme, comprising multiple domains, including the acyltransferase (AT) domain, the acyl-carrier protein (ACP) domain, the ketoacyl synthase (KS) domain, and the TE domain [16].

The β-proteobacterial *Burkholderia* genus is recognized for its significant biosynthetic diversity in natural products, ranking among the top three groups in this regard [17]. This genus holds considerable promise as a source for the development of novel LPs [18,19]. Approximately 30% of the known natural products derived from *Burkholderia* have been discovered using genome mining [20,21]. New LPs can be mined using recombineering system and promoter engineering aimed at activating silent BGCs in *Burkholderia*. For instance, two silent NRPS BGCs (*bgdd* and *hgdd*) on *B. gladioli* ATCC 10248 were activated, leading to the production of new LPs burriogladiodins A–H and haereogladiodins A–B [22]. Additionally, activation of a NRPS/PKS BGC in *B. plantarii* DSM9509 resulted in the discovery of the new LPs haereoplantin F-G [23].

The obligate aerobe, Gram-negative plant pathogen *B. plantarii* DSM9509 (ATCC 43733), isolated from rice seedlings, is a biosafety level 1 microorganism and not pathogenic in humans. In this work, we present the genomic-driven discovery of new natural products in *B. plantarii* DSM9509. Using the recombineering system RecETh1h2e_yi23, which was developed by our team [23], we inserted the constitutive promoter upstream of the *pla* BGC, leading to the identification of a new compound named plantariitin A. This compound exhibits anti-inflammatory activity and is characterized as a linear LP featuring one proteinogenic amino acid alanine (Ala) and two nonproteinogenic amino acids, i.e., homoserine (Hse) and amino-1,2,3,6-tetrahydro-2,6-dioxo-4-pyrimidinepropanoic acid (ATDPP). To the best of our knowledge, this represents the first instance of ATDPP being identified in natural products.

## 2. Results and Discussion

### 2.1. Bioinformatics Analysis of BGC pla

The drugs derived from the natural products of *Burkholderia* are mostly hybrid polyketides and nonribosomal peptides (PKSs/NRPSs) [24,25,26] such as splicostatin [27] and romidepsin [28]. Consequently, our investigation primarily focused on the PKS/NRPS BGCs. Utilizing an antiSMASH analysis of chromosome 2 (GenBank accession no. CP007213.1) of *B. plantarii* DSM9509, we predicted a total of 16 BGCs, including kolossin and plantaribactin, with 100% similarity. BGC 1 (named BGC *pla*) exhibited a relatively low degree of similarity to known BGCs of natural products. This suggests the potential for the production of new natural products with outstanding bioactivity, which could be harnessed for the development of new antitumor or anti-inflammatory drugs.

**Figure 1:**
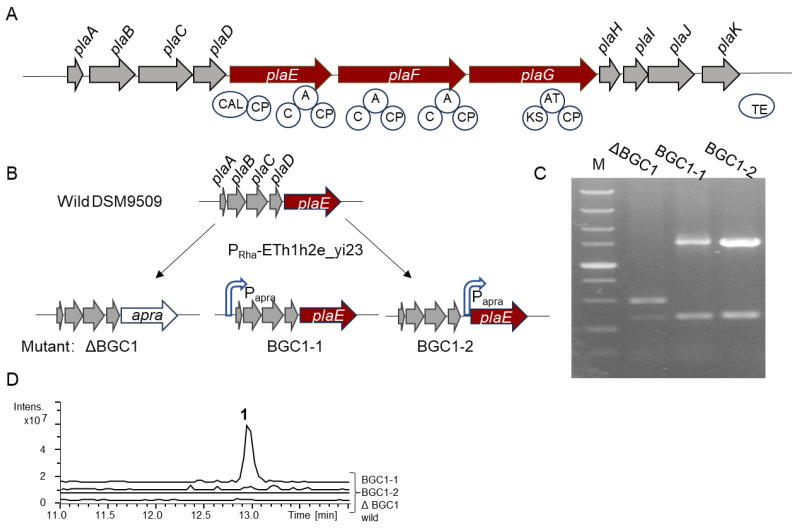
Genome mining of *B. plantarii* DSM9509. (**A**) Schematic drawing of BGC *pla*. CAL, co-enzyme A ligase domain; A, adenylation domain; CP, peptide carrier protein in NRPS or acyl-carrier protein in PKS; C, C domain; and TE, thioesterase domain. NRPSs: *plaE* and *plaF*. PKS gene: *plaG*. (**B**) Construction of mutants via recombineering system RecETh1h2e_yi23. This process shows where apramycin resistance gene (*apra*) or the constitutive promoter (P_apra_) is inserted into the BGC *pla*. (**C**) PCR verification of mutants. M: DL 5000 maker. (**D**) Metabolic profiling of wild strain and mutants. For (**B**–**D**) wild, ΔBGC1, BGC1-1, and BGC1-2 represent *B. plantarii* DSM9509, DSM9509ΔBGC1, DSM9509-BGC1-1, and DSM9509-BGC1-2, respectively.

The 30 kb BGC *pla* contains a total of 11 genes, including two NRPS genes (*plaE* and *plaF*) and one type I PKS gene (*plaG*) (Figure 1A and Table S1). The functional annotations of the remaining genes are shown in Table S1. Bioinformatics analysis revealed that the *plaE* gene comprises five domains: a co-enzyme A ligase domain (CAL), a C domain, an A domain, and two peptidyl carrier protein (PCP) domains, with the A domain specifically recognizing the alanine (Ala) to generate an aminoacyl-adenylate intermediate. The gene *plaF* contains two modules, each containing a C domain, an A domain, and a PCP domain; however, the two A domains are not predicted to recruit their corresponding amino acids. The *plaG* gene consists of three domains: an AT domain, a KS domain, and an acyl-carrier protein (ACP) domain. The AT domain is responsible for the selection and transfer of a carboxylic acid such as acetyl-CoA to the ACP domain. Then, the KS domain catalyzes the condensation reaction to form carbon–carbon bonds between the starter and extender units. This process is further extended by the addition of a carboxylic acid via the KS domain, culminating in the synthesis of a longer fatty acid chain (FA). Therefore, it is anticipated that the BGC *pla* will produce a 3-amino-acid lipopeptide FA-Ala-X_1_-X_2_, where X denotes the amino acids whose specificity cannot be predicted. In addition to the core synthetic genes, this BGC also contains several additional synthase genes, including carbamoyltransferase (*plaA*) and carboxymuconolactone decarboxylase (*plaB*), acylhydrolase (*plaC*), and monooxygenase (*plaH*) (Table S1). As a result, BGC *pla* holds considerable promise for the production of new natural products.

### 2.2. In Situ Activation of BGC pla in B. plantarii DSM9509

The recombineering system RecETh1h2e_yi23 developed by Ruijuan Li facilitated the modification of *B. plantarii* DSM9509 chromosomes [23]. This system requires only a short homologous arm (80 bp) to complete homologous recombination. To investigate the product of BGC *pla* in *B. plantarii* DSM9509, an inactive mutant DSM9509ΔBGC1, was constructed by replacing the core biosynthetic genes with an apramycin resistance gene using the recombineering system RecETh1h2e_yi23 (Figure 1B) and further verified using PCR (Figure 1C). Comparative metabolic profiling of the mutant and the wild-type strain revealed no discernible difference (Figure 1D), indicating that BGC *pla* remains silent under laboratory fermentation conditions. To activate the expression of BGC *pla*, two mutants (DSM9509-BGC1-1 and DSM9509-BGC1-2) were constructed by inserting the constitutive promoter P_apra_ upstream of genes *plaA* or *plaE*, respectively. These mutants were subsequently verified via PCR and sequencing analysis (Figure 1C) and were subjected to fermentation, followed by compound extraction. An analysis using high-performance liquid chromatography–mass spectrometry (HPLC-MS) revealed a new peak (*m*/*z* 554.23 [M + H]^+^) (**1**) in the activation mutant DSM9509-BGC1-1 when compared to the wild-type strain (Figure 1D). These findings indicate that the insertion of the promoter successfully activated the expression of the downstream genes, implicating the synthase genes preceding the core genes in the synthesis of compound **1**. Overall, the results indicate that the silenced BGCs in *Burkholderia* can be activated for expression by using the recombineering system and promoter engineering [22,29]. Furthermore, the promoter insertion location significantly influences the expression of BGCs. Changing the insertion location of the promoter may improve the success rate (50%) of the strategy of activating a cryptic BGC in *B. plantarii* DSM9509 [23]. The limitation of mining gene clusters via in situ activation is that it requires a suitable recombinase to complete the recombination. However, recombinases that complete the intermolecular recombination of DNA are species-specific. This recombineering system (ETh1h2e_yi23), derived from *B. cordobensis* YI23, was applied to the activation of the silent BGCs of *B. glumae* PG1, *B. glumae* DSM9512, and *B. plantarii* 9509, and the construction of a chassis cell of *B. thailandensis* E264 [23,24], so this recombination system is applicable to certain *Burkholderia* species. However, this recombinase may not work in distantly related strains, so researchers should try to optimize the codon of the recombinase gene or find the appropriate recombinase gene through sequence alignment in the distantly related strains.

### 2.3. Identification of the New Lipopeptide (***1***)

**Figure 2:**
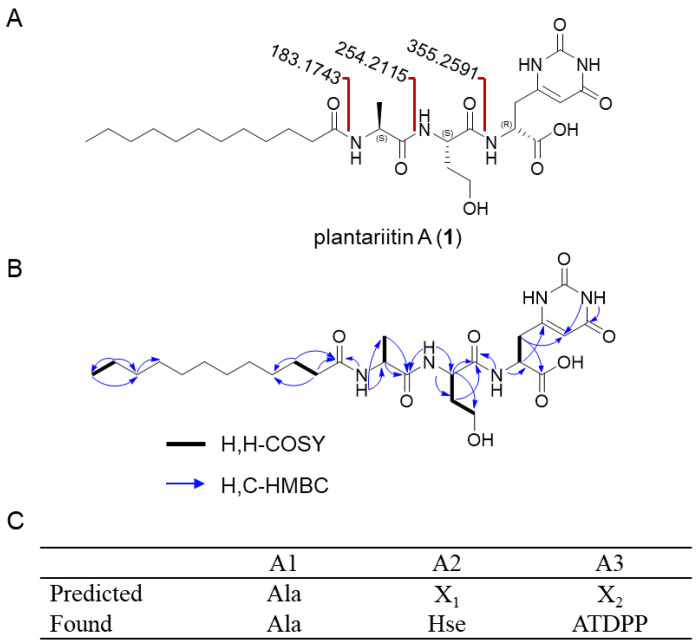
Structure elucidation of plantariitin A (**1**). (**A**) Complete structures of plantariitin A (**1**). Key MS/MS fragments (calculated values 183.17423, 254.2115, 355.2591 [M + H]^+^) showed losses of amino acid residues on the peptidic terminus of **1**. (**B**) Key ^1^H–^1^H COSY (correlated spectroscopy) and HMBC (heteronuclear multiple-bond correlation spectroscopy) of **1**. (**C**) The specificity-conferring code of A domains of BGC *pla*. Ala stands for alanine; Hse stands for homoserine. ATDPP stands for amino-1,2,3,6-tetrahydro-2,6-dioxo-4-pyrimidinepropanoic acid. X_1_ and X_2_ denote amino acids whose specificity cannot be predicted.

The structure of the new lipopeptide (**1**) was fully elucidated via comprehensive analysis using NMR, HR-ESI-MS, MS/MS data, Marfey’s analysis, and NMR calculations (Figure 2A).

Compound **1** was obtained as a white solid powder with the molecular formula deduced to be C_26_H_43_N_5_O_8_ (HRESIMS, *m/z* 554.3196 [M + H]^+^), which suggested 7 indices of hydrogen deficiency. The ^1^H-NMR (Table 1), ^13^C-NMR (Table 1), DEPT-135, and HSQC spectra exhibit 26 carbons, including 7 non-protonated carbons (six carbonyl and one olefinic), 4 methines (one olefinic), 13 methylenes, and 2 methyls. The peptide sequence was determined by analysis of the ^1^H,^1^H-COSY correlations and the HMBC correlations between the amide proton and the adjacent carbonyl group (Figure 2B). This result is also confirmed by the MS/MS fragmentation pattern (Figure S1).

The absolute configurations of Ala and Hse were unambiguously established to be of the L configuration through Marfey’s hydrolysis experiment (Table S2). In contrast, the configuration of ATDPP was determined as the D configuration via quantum chemistry calculations using Gaussian 09 (Figure S8 and Table S3). This non-protein amino acid can be formed in vivo through a series of enzymatic reactions such as L-4-Cl-Kyn [8], a three-step enzyme pathway from L-Trp to L-4-Cl-Kyn in *Streptomyces coelicolor* M1146. We tried to establish the ATDPP synthetase system reaction in *B. plantarii* DSM9509, but unfortunately, we were not successful, and the biosynthesis and function of ATDPP need to be further studied. The introduction of complex amino acids to the peptide chain increases the diversity of LPs and provides new materials for the chemical synthesis of LPs [30].

### 2.4. Anti-Inflammatory Activity of Plantariitin A (***1***)

**Figure 3:**
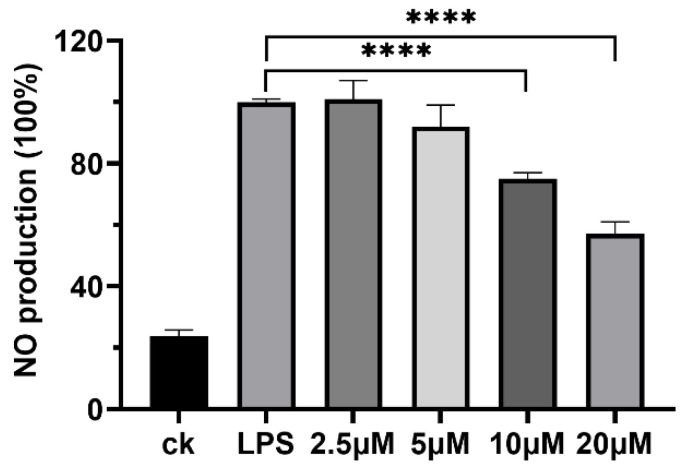
Effects of plantariitin A (**1**) on NO production in RAW264.7 cells stimulated by LPS. CK, NO production in the absence of LPS as the negative control; LPS, 10 μg mL^−1^ LPS-stimulated NO production without compound as the positive control (100%). Error bars, SD; *n* = 3; **** *p* < 0.0001.

Certain LPs, such as polymyxin [31], are recognized for their immunomodulatory activities, which can eliminate inflammatory responses resulting from specific injuries or microbial infection [32,33], thereby indicating their potential as anti-inflammatory agents. In this study, plantariitin A (**1**) was assessed for its capacity to inhibit NO production in lipopolysaccharide (LPS)-induced RAW264.7 cells using the Griess assay. As shown in Figure 3, plantariitin A (**1**) showed inhibition of NO generation activity at 10 and 20 μM. Furthermore, it exhibited no inhibitory effects on lung cancer cell A549, with an IC_50_ greater than 20 μM. Currently, the majority of LPs derived from *Burkholderia* are noted for their remarkable biosurfactant and antimicrobial properties [4,34]. There is a paucity of research focusing on the anti-inflammatory properties of LPs, especially linear LPs.

## 3. Materials and Methods

### 3.1. Materials

The strains and plasmids used in this study are listed in Table S4. *B. plantarii* DSM9509 was ordered from the Leibniz Institute DSMZ. Plasmid pBBR1-Rha-ETh1h2e_yi23-kan contains recombinase operons that were used to edit the genome. Oligonucleotides were synthesized by Sangon Biotech Co., Ltd., Shanghai, China (Table S5). *Burkholderia* species were cultured at 30 °C in CYMG medium (4 g L^−1^ yeast extract, 8 g L^−1^ casein peptone, 5 mL L^−1^ glycerin, 8.06 g L^−1^ MgCl_2_·6H_2_O). Antibiotics were added as required. The concentration of apramycin (apra) was 250 μg mL^−1^; the concentration of kanamycin (kan) was 30 μg mL^−1^.

### 3.2. Construction of Mutants

The BGC *pla* activation mutants were constructed by inserting the promoter (P_apra_) of the apramycin antibiotic resistance gene to replace the original promoter upstream of genes *plaA* or *plaE*. The target DNA fragments with an 80 bp homologous arm were amplified via PCR with plasmid p15A-cm-apra used as a template. Primers used in this study are listed in Table S5. For recombineering, the recombinase expression plasmid pBBR1-Rha-ETh1h2e_yi23-kan was electroporated into *B. plantarii*. The recombineering was performed as described previously [23]. Correct recombinants were selected on CYMG medium containing 250 μg mL^−1^ apramycin and verified using colony PCR.

### 3.3. Fermentation, Extraction, and Isolation

Seed cultures of *B. plantarii* DSM9509-BGC1-1 diluted at a ratio of 1:50 were fermented in 1.5 L flash CYMG medium at 30 °C, 180 rpm for 2 days. Then, 2% Amberlite XAD-16 (*v*/*v*) was added followed by incubation for 12 h. The XAD-16 and biomass were collected at a speed of 7000 rpm for 20 min centrifugation and extracted with 500 mL methanol. After they were soaked in methanol for 6 h, the methanol and XAD-16 were separated using filter paper. Finally, the methanol was dried using a rotary steamer.

A 15 L fermentation solution was prepared and dissolved in 50 mL of methanol for the separation of compound **1**. The final methanol extracts were separated into several fractions on a silica gel column using step-gradient elution with CH_2_Cl_2_ and MeOH (50:1 to 1:1). The fractions containing compound **1** were further purified using semi-preparative reverse-phase HPLC (ODS; Bruker ZORBAX SB-C18 (Billerica, MA, USA), 5 μm, 250 × 10 mm, 2 mL min^−1^). The acetonitrile (ACN) and H_2_O containing 0.1% trifluoroacetic acid were used as the mobile phase A and B, respectively, under the conditions 0−4 min, 30% ACN; 4−28 min, 30−90% ACN; 28.1 min, 95% ACN; and 28.1−32 min, 95% ACN to obtain plantariitin A (**1**) (5.2 mg, t_R_ = 17.5 min).

Plantariitin A (**1**): UV (MeOH) *λ_max_* (logε) 262 (1.74) nm (Figure S9); IR (3285, 2937, 2861, 1667, 1538) cm^−1^ (Figure S10); ^1^H and ^13^C NMR (Table 1).

### 3.4. Determination of the Amino Acid Ala and Hse Configuration

Approximately 1 mg plantariitin A was hydrolyzed with 6 M HCl (600 μL) at 60 °C for 24 h. The solution was dried and was dissolved in 200 μL H_2_O. At the same time, relevant standard amino acids (1 mg) were also dissolved in 200 μL H_2_O. Then, 1 M NaHCO_3_ (25 µL) and 2.5 µM L-FDAA (200 μL) were added to the above sample. The sample was mixed and incubated for 1 h at 40 °C. Then, 2 M HCl (25 µL) was added to quench the reaction. Next, the mixture was separated at 7000 rpm for 2 min centrifugation and analyzed via HPLC-MS as described previously [22].

### 3.5. Determination of the Amino Acid ATDPP Configuration

The theoretical calculations in respect of plantariitin A were performed using Gaussian 09. First, the possible conformations obtained using Spartan’14 software were optimized at the B3LYP/6-31G* level in the gas phase. Then, based on the Boltzmann distribution law, the room-temperature equilibrium populations were calculated. NMR calculations were performed using the GIAO method [35] at the MPW1PW91/6-311G**//B3LYP/6-31G* level in DMSO-*d*_6_ with the PCM model. The shielding constants (including ^13^C and ^1^H) obtained and the DP4+ probability were used for statistical analyses of experimental chemical shifts directly [36].

### 3.6. Detection of NO Production

RAW264.7 cells were inoculated into 96-well plates with 5% CO_2_ at 37 °C for 24 h. Compound **1** (2.5, 5, 10, and 20 μM) was added to pretreat cells for 30 min. Then, lipopolysaccharides (10 μg mL^−1^) were added and cultured for 24 h, and 50 μL of supernatant was extracted for nitric oxide detection using a nitric oxide assay kit with Griess reagents (Beyotime, Lot: S0021, Shanghai, China) at room temperature. The absorbance value was detected using a microplate reader at 540 nm.

## 4. Conclusions

The discovery of new natural products derived from *Burkholderia* has significantly increased, attributed to advancements in recombination engineering and promoter engineering. In this study, the silent NRPS/PKS BGC *pla* was successfully activated in *B. plantarii* DSM9509. A structural analysis revealed that the compound named plantariitin A is a new LP comprising three amino acids. Notably, plantariitin A shows low structural similarity to existing natural products and is distinguished by the presence of an unusual nonproteinogenic amino acid residue, amino-1,2,3,6-tetrahydro-2,6-dioxo-4-pyrimidinepropanoic acid, which has not been identified in other NRPSs or NRPS/PKS-derived compounds. Furthermore, plantariitin A demonstrates anti-inflammatory activity. In summary, the discovery of new natural products has been greatly enhanced by the activation of silenced gene clusters and the mining of the genome.

## Figures and Tables

**Table 1:** ^1^H (600 MHz) and ^13^C NMR (125 MHz) data in respect of **1** in DMSO-*d*_6_.

	No.	δ_C_		δ_H_ (*J* in Hz)
dodecanoic acid	1	172.3	C	
	2	35.1	CH_2_	2.08 (td, 10.2, 2.4)
	3	25.2	CH_2_	1.46 (m)
	4	28.8	CH_2_	1.23 (m ^a^)
	5	29.1	CH_2_	1.23 (m ^a^)
	6	29.1	CH_2_	1.23 (m ^a^)
	7	29.0	CH_2_	1.23 (m ^a^)
	8	28.9	CH_2_	1.23 (m ^a^)
	9	28.7	CH_2_	1.23 (m ^a^)
	10	31.3	CH_2_	1.22 (m ^a^)
	11	22.1	CH_2_	1.26 (m)
	12	14.0	CH_3_	0.85 (t, 7.2)
L-Ala	1	172.4	C	
	2	48.1	CH	4.24 (p, 7.2)
	3	18.0	CH_3_	1.15 (d, 7.2)
			NH	7.94 (d, 7.8)
L-Hse	1	171.6	C	
	2	50.0	CH	4.27 (m)
	3	35.1	CH_2_	1.80 (m); 1.63 (m)
	4	57.5	CH_2_	3.38 (m ^a^)
			NH	7.91 (d, 9.0)
D-ATDPP	1	171.9	C	
	2	49.9	CH	4.50 (m)
	3	33.7	CH_2_	2.78 (dd, 14.4, 5.4); 2.61 (dd, 14.4, 8.4)
	4	152.3	C	
	5	99.7	CH	5.28 (s)
	6	164.0	C	
	6a		NH	10.89 (brs)
	8	151.6	C	
	8a		NH	10.89 (brs)
			NH	8.09 (m)

^a^ overlap.

## Data Availability

The data presented in this study are available in the article and Supplementary Materials.

## Supplementary Materials

The following supporting information can be downloaded at: https://www.mdpi.com/article/10.3390/molecules30040868/s1, Table S1. Predicted gene function of BGC *pla*; Table S2. Retention time of amino acids after derivatization with Marfey’s reagent; Table S3. Experimental chemical shifts in plantariitin A, calculated shielding tensors and chemical shifts in isomers **1** and **2** in DMSO with TMS as a reference at the MPW1PW91/6-311G** level; Table S4. Strains, mutants, and plasmids in this work; Table S5. Oligonucleotide sequences used in this study; Figure S1. MS/MS fragmentation analysis and spectrum of **1**; Figure S2. ^1^H NMR spectrum (600 MHz) of **1** in DMSO-*d*_6_; Figure S3. ^13^C NMR spectrum (150 MHz) of **1** in DMSO-*d*_6_; Figure S4. DEPT135 spectrum (150 MHz) of **1** in DMSO-*d*_6_; Figure S5. HSQC spectrum (600 MHz) of **1** in DMSO-*d*_6_; Figure S6. ^1^H-^1^H COSY spectrum (600 MHz) of **1** in DMSO-*d*_6_; Figure S7. HMBC spectrum (600 MHz) of **1** in DMSO-*d*_6_; Figure S8. Detailed DP4+ probability of compound **1** calculated at the mPW1PW91/6-311G** level in DMSO with the PCM model; Figure S9. UV spectrum of compound **1**; Figure S10. IR spectrum of compound **1**; Figure S11. CD spectrum of compound **1**.

## Abbreviations

BGCs	biosynthetic gene clusters
LPs	lipopeptides
NRPSs	non-ribosomal peptide synthetases
PKSs	polyketide synthases
FA	fatty acid chain
Ala	alanine
Hse	homoserine
ATDPP	amino-1,2,3,6-tetrahydro-2,6-dioxo-4-pyrimidinepropanoic acid
NMR	nuclear magnetic resonance
HR-ESI-MS	high-resolution electrospray ionization mass spectrometry
DEPT	distortionless enhancement via polarization transfer
HSQC	heteronuclear single quantum coherence
COSY	correlated spectroscopy
HMBC	heteronuclear multiple-bond correlation spectroscopy
