# Supplementary material for: Genomics-Driven Discovery of Plantariitin A, a New Lipopeptide in Burkholderia plantarii DSM9509
Source: Molecules. 2025 Feb 14;30(4):868. doi: 10.3390/molecules30040868 (PMC11858073; doi:10.3390/molecules30040868)
Supplement: Supplementary file 1 [file molecules-30-00868-s001.zip › molecules-3454800-supplementary.pdf]

Supplementary Materials for

Genomics-Driven Discovery of Plantariitin A, a New Lipopeptide in *Burkholderia plantarii* DSM9509

Xiuling Wang, Zhuo Zhang, Jun Fu*, and Ruijuan Li*

State Key Laboratory of Microbial Technology, Shandong University,

Qingdao 266237, China

*Correspondence should be addressed to Jun Fu (fujun@sdu.edu.cn) or Ruijuan Li (liruijuan@sdu.edu.cn).

Tel. +86-532-67722918 (J.F. & R.L.); Fax +86-532-58631501 (J.F. & R.L.).

Supplementary Materials for Tables and Figures

Table S1. Predicted gene function of BGC *pla*.

Table S2. Retention time of amino acids after derivatization with Marfey's reagent.

Table S3. Experimental chemical shifts of plantariitin A, calculated shielding tensors and chemical shifts of isomers **1** and **2** in DMSO with TMS as reference at MPW1PW91/6-311G** level.

Table S4. Strains, mutants and plasmids in this work.

Table S5. Oligonucleotide sequences used in this study.

Figure S1. MS/MS fragmentation analysis and spectrum of **1**.

Figure S2. ^{1}H NMR spectrum (600 MHz) of **1** in DMSO-d_6.

Figure S3. ^{13}C NMR spectrum (150 MHz) of **1** in DMSO-d_6.

Figure S4. DEPT135 spectrum (150 MHz) of **1** in DMSO-d_6.

Figure S5. HSQC spectrum (600 MHz) of **1** in DMSO-d_6.

Figure S6. ^{1}H-^{1}H COSY spectrum (600 MHz) of **1** in DMSO-d_6.

Figure S7. HMBC spectrum (600 MHz) of **1** in DMSO-d_6.

Figure S8. Detailed DP4+ probability of compound **1** calculated at mPW1PW91/6-311G** level in DMSO with PCM model.

Figure S9. UV spectrum of compound **1**.

Figure S10. IR spectrum of compound **1**.

Figure S11. CD spectrum of compound **1**.

Table S1. Predicted gene function of BGC *pla*.

Gene	Identifier	Predicted protein function
plaA	bpln_RS18365	carboxymuconolactone decarboxylase family protein
plaB	bpln_RS18360	carbamoyltransferase N-terminal domain-containing protein
plaC	bpln_RS18355	GDSL-like lipase/acylhydrolase protein
plaD	bpln_RS18350	MFS transporter
plaE	bpln_RS18345	non-ribosomal peptide synthetase
plaF	bpln_RS18340	non-ribosomal peptide synthetase
plaG	bpln_RS18335	type I polyketide synthase
plaH	bpln_RS18330	MupA/Atu3671 family FMN-dependent luciferase-like monooxygenase
plaI	bpln_RS18325	hypothetical protein
plaJ	bpln_RS18320	penicillin acylase family protein
plaK	bpln_RS18315	NAD (+) synthase

Table S2. Retention time of amino acids after derivatization with Marfey's reagen.

Amino acid (aa)	Configuration	Retention time (min)	
		Standard aa	**1**
Ala	L	10.41	10.42
	D	10.95	
Hse	L	9.55	9.54
	D	9.68	

Table S3. Experimental chemical shifts of plantariitin A calculated shielding tensors and chemical shifts of isomers **1** and **2** in DMSO with TMS as reference at MPW1PW91/6-311G** level.

nulei	num.	δ (exptl) plantariitin A	σ iso-isomer **1**	δ iso-**1** (calcd)	δ iso-**1** (scaled)	σ iso-isomer **2**	δ iso-**2** (calcd)	δ iso-**2** (scaled)
C	11	22.1	162.3	26.2	22.3	160.4	28.1	25.1
C	10	31.3	154.6	33.9	29.7	153.1	35.3	32.2
C	9	28.7	157.6	30.9	26.8	153.7	34.8	31.6
C	8	28.9	156.0	32.5	28.3	156.7	31.8	28.7
C	7	29.0	154.1	34.3	30.1	159.5	29.0	26.1
C	6	29.1	159.5	29.0	24.9	156.9	31.6	28.5
C	5	29.1	154.6	33.9	29.7	158.6	29.9	26.9
C	4	28.8	155.8	32.7	28.5	158.1	30.3	27.3
C	3	25.2	157.8	30.7	26.6	158.9	29.5	26.6
C	2	35.1	146.3	42.1	37.6	147.3	41.2	37.8
C	1	172.3	2.0	186.5	176.5	4.3	184.1	175.6
C	2	48.1	128.0	60.5	55.3	133.8	54.7	50.8
C	1	172.4	2.8	185.7	175.7	5.4	183.1	174.5
C	2	50.0	129.2	59.2	54.1	134.2	54.3	50.5
C	1	171.6	6.0	182.5	172.6	6.3	182.2	173.7
C	2	49.9	129.6	58.8	53.7	133.0	55.5	51.5
C	1	171.9	10.2	178.3	168.6	5.5	183.0	174.5
C	3	18.0	170.7	17.8	14.2	168.1	20.4	17.8
C	3	35.1	153.5	35.0	30.7	158.5	30.0	27.0
C	4	57.5	128.4	60.1	54.9	127.0	61.5	57.4

C	3	33.7	146.3	42.2	37.7	150.0	38.5	35.2
C	4	152.3	25.4	163.1	154.0	29.9	158.5	150.9
	5	99.7	81.5	107.0	100.0	82.5	106.0	100.3
H	6	164.0	21.4	167.1	157.8	22.3	166.1	158.2
H	8	151.6	31.6	156.9	148.0	34.1	154.4	146.9
H	12	14.0	173.9	14.6	11.1	172.3	16.1	13.7
H				-	-		-	-
H	11	1.26	30.50	1.29	1.25	31.05	0.75	0.81
H	11	1.26	30.70	1.09	1.05	30.77	1.02	1.05
H	10	1.22	30.65	1.14	1.10	30.58	1.21	1.22
H	10	1.22	30.56	1.24	1.20	30.94	0.86	0.90
H	9	1.23	30.47	1.32	1.28	30.66	1.14	1.16
H	9	1.23	30.82	0.98	0.94	30.71	1.08	1.11
H	8	1.23	30.61	1.19	1.15	30.96	0.84	0.89
H	8	1.23	30.57	1.23	1.18	30.43	1.37	1.36
H	7	1.23	30.94	0.86	0.82	30.51	1.28	1.29
H	7	1.23	30.38	1.42	1.37	30.59	1.21	1.22
H	6	1.23	30.79	1.00	0.97	30.41	1.38	1.38
H	6	1.23	30.42	1.38	1.33	30.44	1.36	1.35
H	5	1.23	30.66	1.13	1.09	30.61	1.19	1.20
H	5	1.23	30.48	1.31	1.27	30.48	1.32	1.32
H	4	1.23	30.33	1.47	1.42	30.55	1.24	1.25
H	4	1.23	30.53	1.26	1.22	30.85	0.94	0.98
H	3	1.47	29.97	1.82	1.77	30.49	1.31	1.31
H	3	1.47	30.38	1.42	1.37	29.81	1.99	1.92

H	2	2.08	29.77	2.03	1.97	29.73	2.07	1.99
H	2	2.08	29.71	2.08	2.02	29.23	2.57	2.44
H	2	4.24	28.18	3.62	3.53	27.29	4.51	4.19
H	2	4.27	27.81	3.98	3.88	27.07	4.73	4.38
H	2	4.50	27.08	4.71	4.59	26.93	4.86	4.51
H	3	1.15	30.06	1.74	1.69	30.38	1.41	1.40
H	3	1.80	29.76	2.03	1.97	29.57	2.23	2.14
H	3	1.63	30.06	1.74	1.68	30.04	1.75	1.71
H	4	3.38	28.32	3.48	3.39	27.78	4.02	3.75
H	4	3.38	27.50	4.30	4.19	28.16	3.64	3.40
H	3	2.78	29.28	2.51	2.44	28.85	2.95	2.79
H	3	2.61	28.72	3.07	2.99	29.37	2.42	2.31
H	5	5.28	26.05	5.74	5.61	26.38	5.42	5.00
H	12	0.85	30.82	0.97	0.94	30.89	0.90	0.95

Table S4. Strains, mutants and plasmids in this work.

Strains	Genotype	Source
B. Plantarii		
DSM9509	Wild-type	DSMZ
DSM9509ΔBGC1	The fragment (132328–137737) of BGC1 was replaced by apramycin resistance gene in DSM9509	This study
DSM9509-BGC1-1	The fragment (143772–143931) of BGC1 was replaced by apramycin resistance gene in DSM9509	This study
DSM9509-BGC1-2	The fragment (137737–137863) of BGC1 was replaced by apramycin resistance gene in DSM9509	This study
Plasmids		
pBBR1-Rha-ETh1h2e_yi23-kan	pBBR1 replicon, kan^R, recombinase ETh1h2e_yi23 under the control of rhamnose promoter	Our lab [1]
p15A-cm-apra	p15A replicon, cm^R, $apra^R$	Our lab

Table S5. Oligonucleotide sequences used in this study.

Primers	Primer sequences (5’-3’)
Δ1-1	gccgcctgcgcggtgctgggctgggtcgcgctgcgccggcacccggtcctgtcgcggcagtccgatcgcgccacctgaACGCTCAGTGGAACGAGGTTC
Δ1-2	gcgccgccagtaacgtgacgtaggcggccaggtcggccaccgtggtgcgctccagcaccttggccagggagatttcgacgCATAATCTGTACCTCCTTA
DΔ1-1	CACCGTGTTCGGCATGCTC
DΔ1-2	CCAAGTGGCCCATCTTCGAG
1-1	tcggaattgccaattccaattcgatcgcgattcgatccaacctttccgattcatttcacgctgaaattcgaggcgcgcgcACGCTCAGTGGAACGAGGTTC
1-2	gagtaatgccagacctggtgcttctcgtcggcgttgcaggagcgcaattccggtatcgagcggtaattgaaataaatcatCATAATCTGTACCTCCTTA
D1-1	AACCGAGAGTCGGCAGTCG
D1-2	AGCAGCAACTGGTAGAACAG
2-1	gccgcctgcgcggtgctgggctgggtcgcgctgcgccggcacccggtcctgtcgcggcagtccgatcgcgccacctgaACGCTCAGTGGAACGAGGTTC
2-2	atcgtcgcgctccggtgcggcttcgaggtaattcgcttggaaagggaagggcgttcgcgggccgcttgcgcgggccgcaTCATAATCTGTACCTCCTTA
D2-1	CACCGTGTTCGGCATGCTC
D2-2	TCGAGTTCGGCGAAGCTCAG

Lower case letters are homology arms.

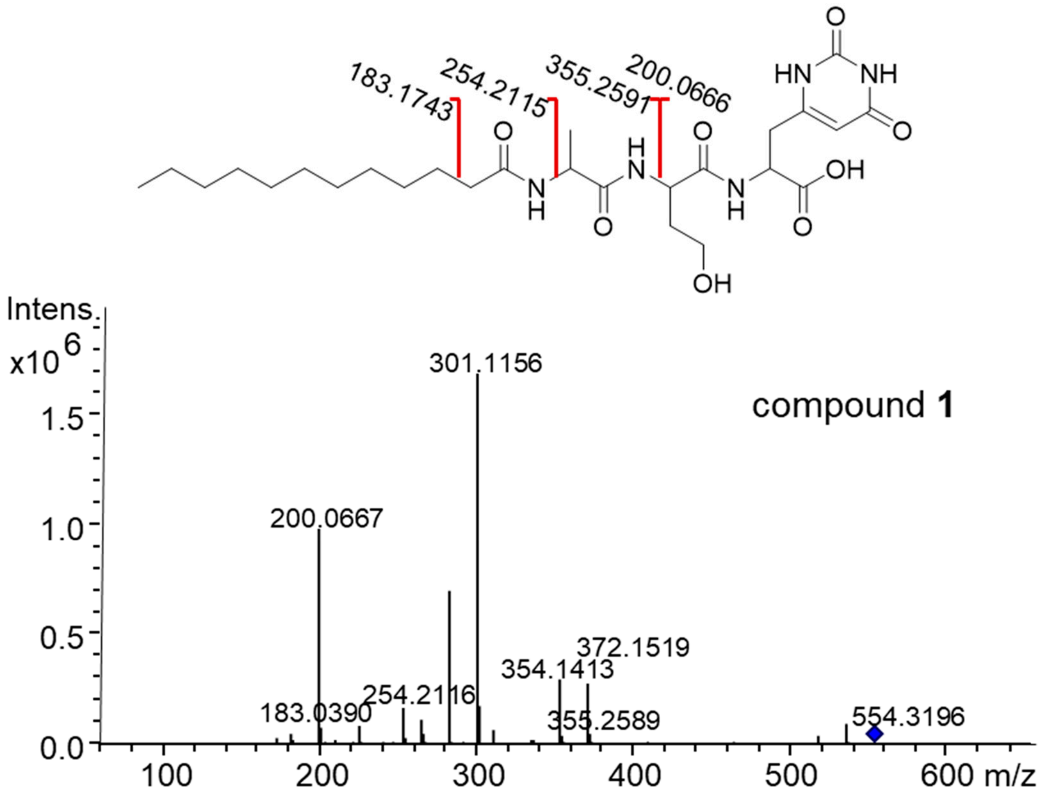

Figure S1. MS/MS fragmentation analysis and spectrum of **1**.

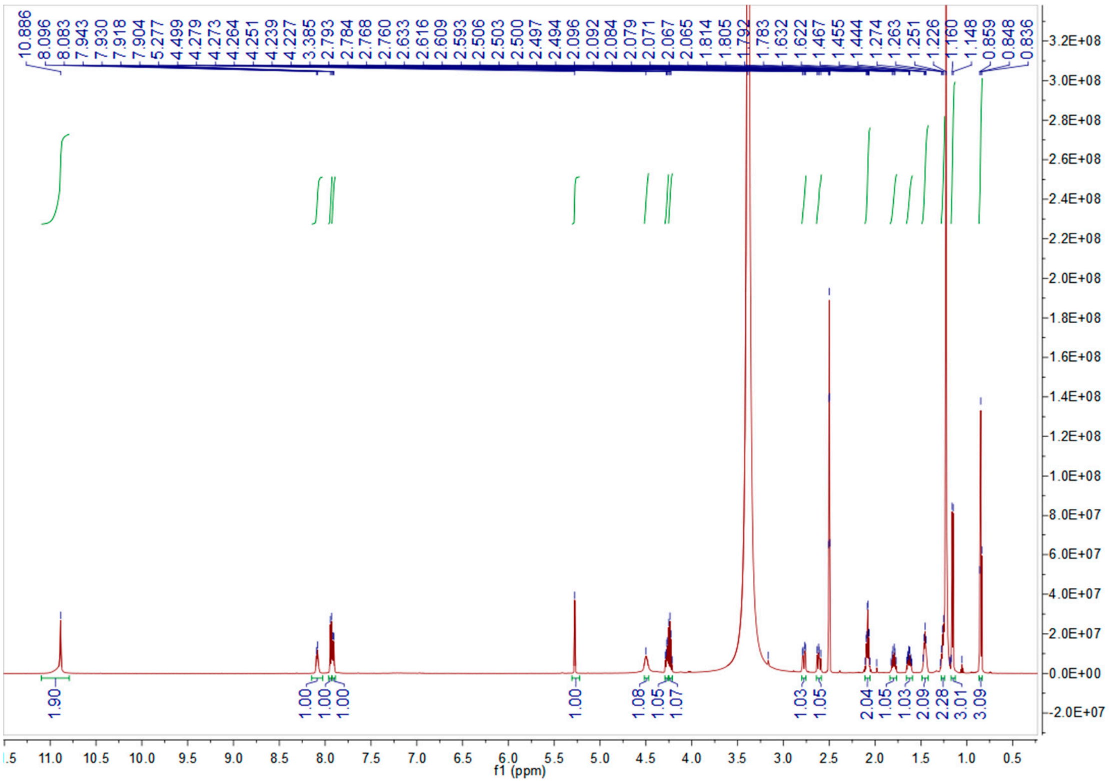

Figure S2. ^{1}H NMR spectrum (600 MHz) of **1** in DMSO-d_6.

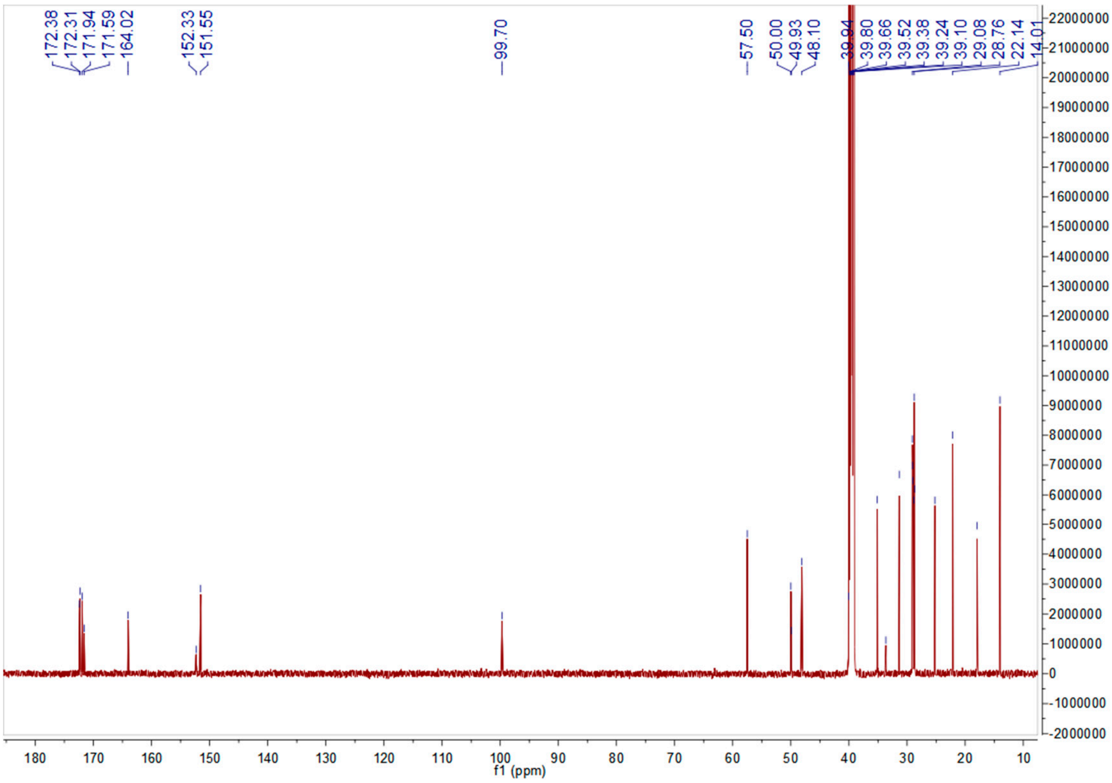

Figure S3. ^{13}C NMR spectrum (150 MHz) of **1** in DMSO-d_6.

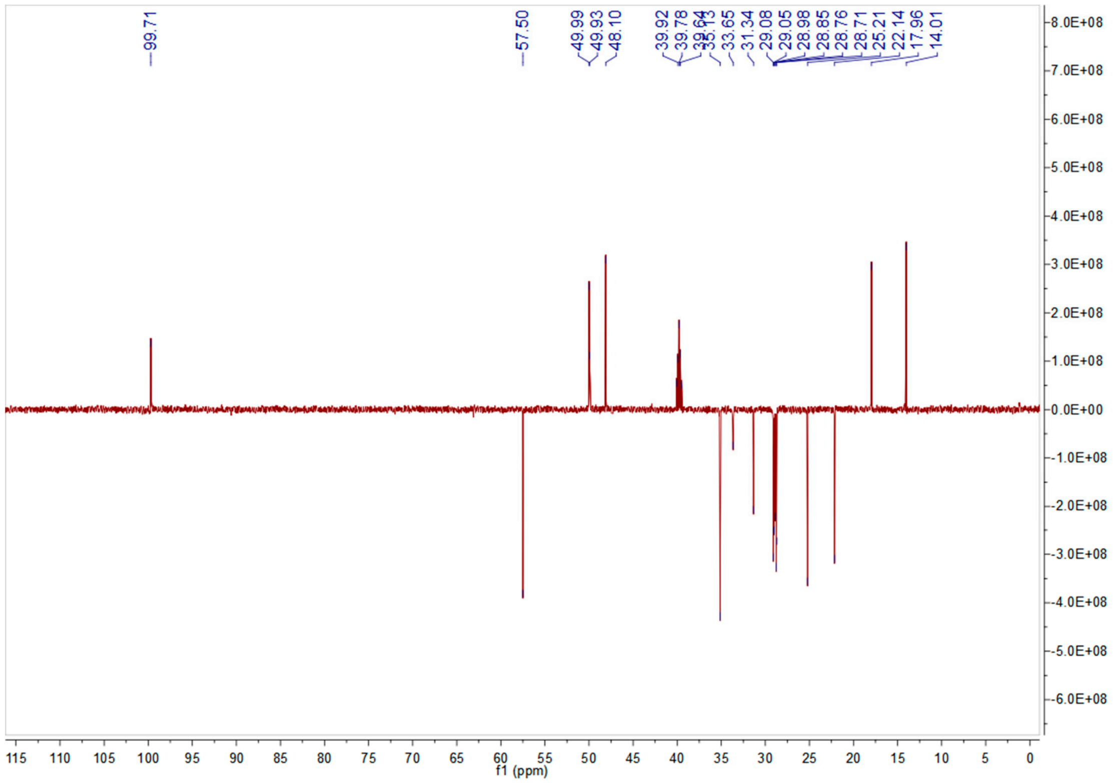

Figure S4. DEPT135 spectrum (150 MHz) of **1** in DMSO-d_6.

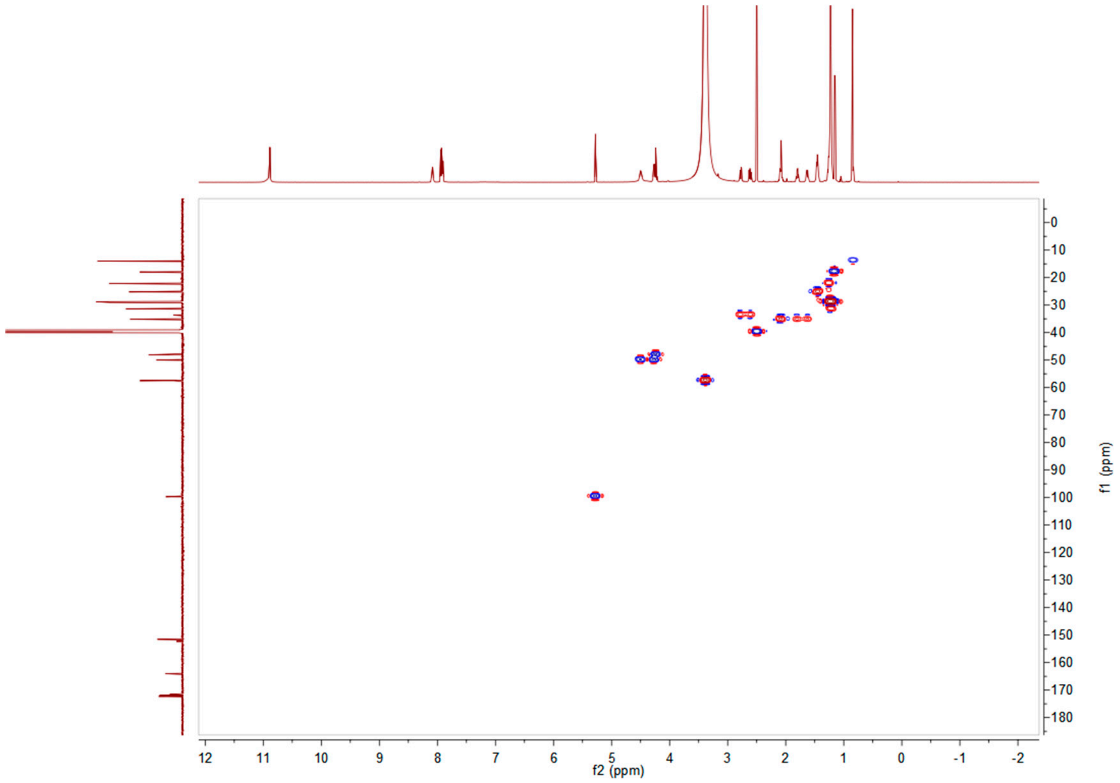

Figure S5. HSQC spectrum (600 MHz) of **1** in DMSO-d_6.

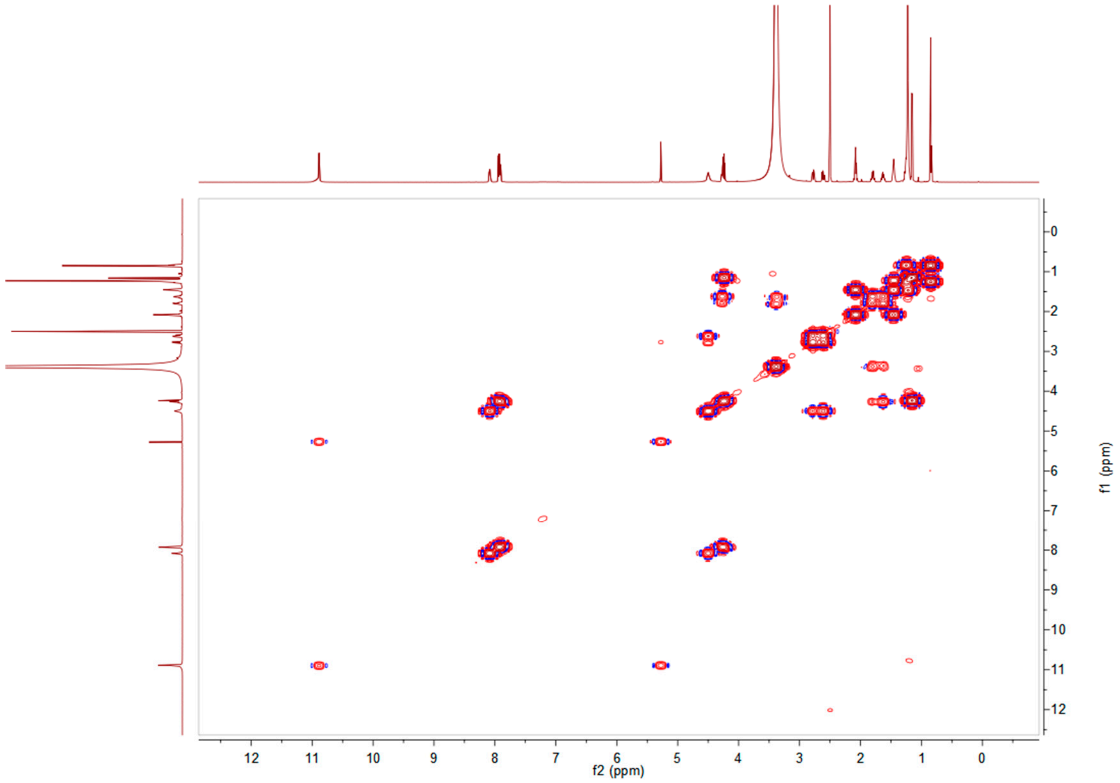

Figure S6. ^1H-^1H COSY spectrum (600 MHz) of **1** in DMSO-*d*6.

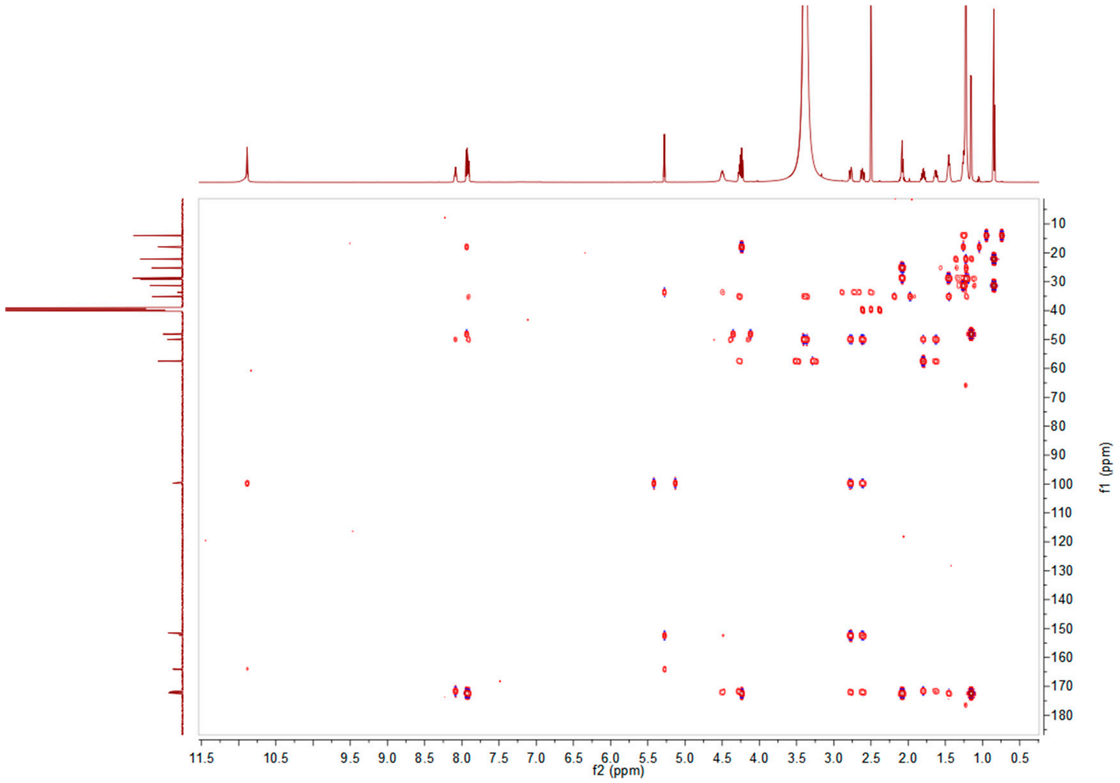

Figure S7. HMBC spectrum (600 MHz) of **1a** in DMSO-*d*6.

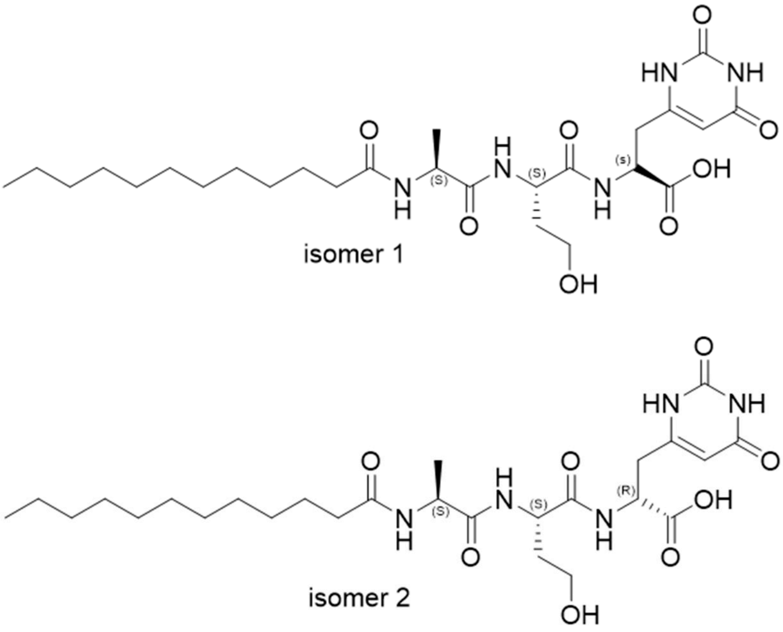

Settings		Type of data (shifts)		
Default		Shielding tensors		
**Functional**		**Solvent?**	**Basis Set**	
mPW1PW91		PCM	6-311G(d,p)	
**Isomer №**		**1**	**2**	**3**
**DP4+ (%)**	**H data**	42.89%	57.11%	-
	**C data**	0.00%	100.00%	-
	**All data**	0.00%	100.00%	-

Figure S8. Detailed DP4+ probability of compound **1** calculated at mPW1PW91/6-311G** level in DMSO with PCM model.

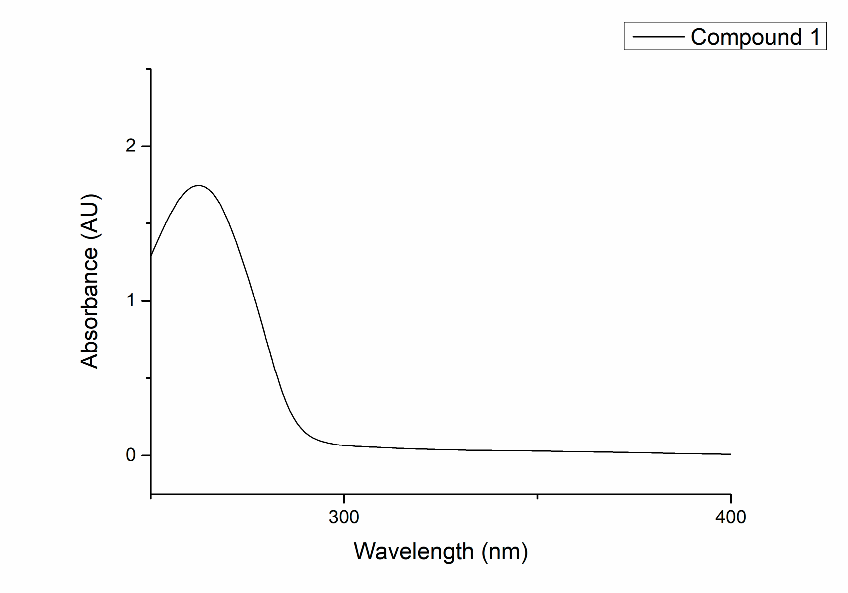

Figure S9. UV spectrum of compound **1**.

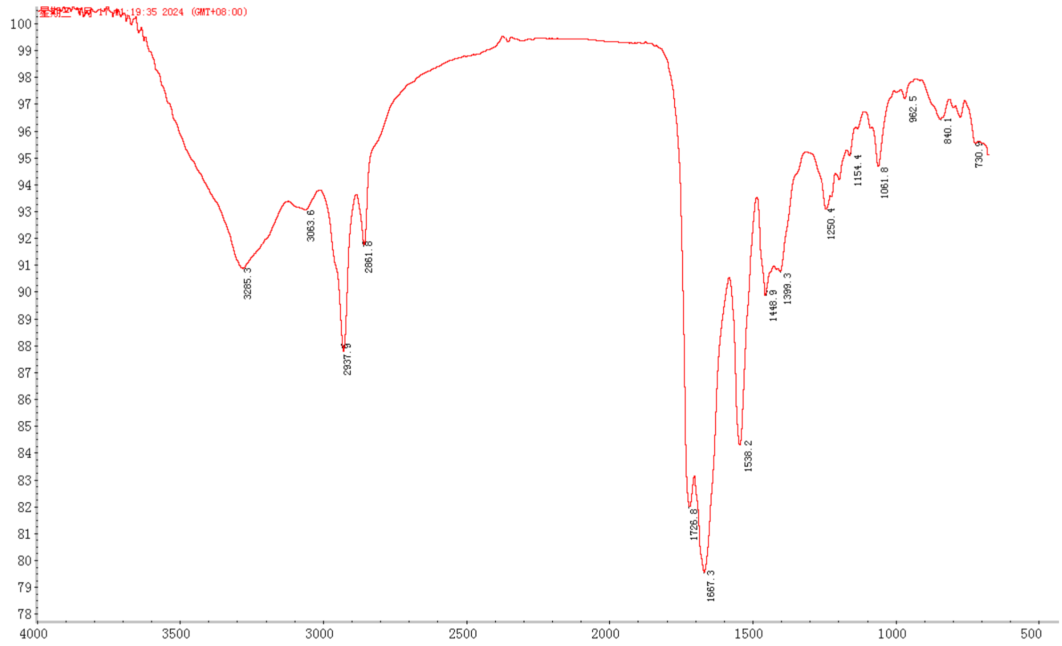

Figure S10. IR spectrum of compound **1**.

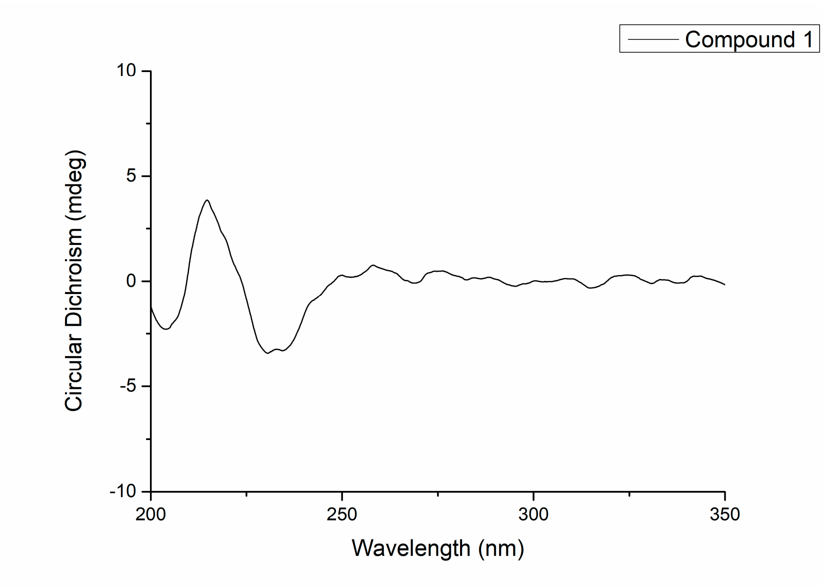

Figure S11. CD spectrum of compound **1**.

Reference

[1] Li, R.; Shi, H.; Zhao, X.; Liu, X.; Duan, Q.; Song, C.; Chen, H.; Zheng, W.; Shen, Q.; Wang, M.; et al. Development and application of an efficient recombineering system for *Burkholderia glumae* and *Burkholderia plantarii*. *Microb. Biotechnol.* **2021**, *14*, 1809–1826.
